# Supplementary material for: Follow-up of a historic cohort of children treated for severe acute malnutrition between 1988 and 2007 in Eastern Democratic Republic of Congo
Source: PLoS One. 2020 Mar 11;15(3):e0229675. doi: 10.1371/journal.pone.0229675 (PMC7065746; doi:10.1371/journal.pone.0229675)
Supplement: Attachment — Submitted filename: Response to Reviewers (FV).docx [file pone.0229675.s001.docx]

A. **Journal Requirements:**

***I would like to update my funding statement.***

*Currently, my Funding Statement reads as follows: "The funders had no role in study design, data collection and analysis, decision to publish, or preparation of the manuscript." Instead of that, please put "This study is part of a Research for the Development Project entitled: ‘Implementation study of a psycho-medico-social care model at the health centre level: the case of people with chronic diseases and the mother-child malnourished couple, South Kivu, Democratic Republic of Congo’ and funded by Belgian Development Cooperation through the Académie de Recherche et d’Enseignement Supérieur (ARES). The funder of the study had no role in study design, data collection, data analysis, data interpretation, or writing of the report. "*

B. **Reviewers' comments:**

**1.Are there data on readmissions of patients for SAM over the years other than the incident admissions?**

R/ *At this time, we do not have data on readmissions of patients identified for SAM during their childhood. Given the objective and scope of our study and due to financial and logistical constraints we could not check in all the health facilities in the region if these children were readmitted for SAM. However, among the causes of death in our possession, we observed that around 14% of deaths were secondary to a relapse of SAM. But, the long-term effects of SAM would likely also be influenced by the numbers of hospitalizations. In our future studies, by studying the effects in relation to the subgroups, we believe that this information will be of use to justify certain differences probably observed.*

**2.Is it known and can be analysed the impact of medical campaigns of SAM? And data of social context where each individual was going back to after discharge?**

R/

*-It is very difficult for us to know and especially to analyze the possible impact of medical campaigns on SAM over such a long period retrospectively due to data unavailable*

*-In terms of social context, the vast majority of this population lives in a very difficult socio-economic situation, as mentioned in the introduction. We do not have individual data from the specific context in which the children lived after nutritional rehabilitation. Nevertheless, the fact that we have observed long-term persistent effects of SAM during childhood on growth in adulthood is a sufficient proof that they continued to live in the same unfavorable conditions even after nutritional rehabilitation. Furthermore, given their age at admission and the fact that most of them live outside the parental home at the moment, it is difficult for us to say exactly the social context in which they lived after nutritional rehabilitation. However, we will describe in a future study their current socioeconomic context.*

**3.It is suggested to structure the results section into paragraphs.**

R/ *Thank you for the remark. It has been taken into account and incorporated in the text.*

**4.It will be interesting to note if possible the presence of alcohol abuse and associated psychiatric disorders.**

R/ *We have data on alcohol consumption in our population study but not on alcohol abuse. However, based on the objective and scope of our work, we opted to use this information for the next studies in which we will examine the risk of cardio-metabolic pathologies in depth. Concerning psychiatric disorders, we have no information now. However, we are conducting a study in which we are measuring the cognitive development of our subjects in adulthood which will allow us to integrate certain tests including the Mini Mental Statu Examination (MMSE) in order to detect cognitive disorders and hence request information related to the psychiatric disorders.*
